# Costs of delivering human papillomavirus vaccination to schoolgirls in Mwanza Region, Tanzania

**DOI:** 10.1186/1741-7015-10-137

**Published:** 2012-11-13

**Authors:** Wilm Quentin, Fern Terris-Prestholt, John Changalucha, Selephina Soteli, W John Edmunds, Raymond Hutubessy, David A Ross, Saidi Kapiga, Richard Hayes, Deborah Watson-Jones

**Affiliations:** 1 Department of Health Care Management, Berlin University of Technology, Straße des 17 Juni 135, Berlin, 10623, Germany; 2 Department of Global Health and Development, London School of Hygiene and Tropical Medicine, 15-17 Tavistock Place, London, WC1H 9SH, UK; 3 Mwanza Research Centre, National Institute for Medical Research, PO Box 1462, Mwanza, Tanzania; 4 Mwanza Intervention Trials Unit, National Institute for Medical Research, PO Box 11936, Mwanza, Tanzania; 5 Department of Infectious Disease Epidemiology, London School of Hygiene and Tropical Medicine, Keppel Street, London, WC1E 7HT, UK; 6 Initiative for Vaccines Research, World Health Organization, Avenue Appia, 1211 Geneva 27, Switzerland; 7 Department of Clinical Research, London School of Hygiene and Tropical Medicine, Keppel Street, London, WC1E 7HT, UK

**Keywords:** Africa, costs and cost analysis, economics papillomavirus vaccines, uterine cervical neoplasms

## Abstract

**Background:**

Cervical cancer is the leading cause of female cancer-related deaths in Tanzania. Vaccination against human papillomavirus (HPV) offers a new opportunity to control this disease. This study aimed to estimate the costs of a school-based HPV vaccination project in three districts in Mwanza Region (NCT ID: NCT01173900), Tanzania and to model incremental scaled-up costs of a regional vaccination program.

**Methods:**

We first conducted a top-down cost analysis of the vaccination project, comparing observed costs of age-based (girls born in 1998) and class-based (class 6) vaccine delivery in a total of 134 primary schools. Based on the observed project costs, we then modeled incremental costs of a scaled-up vaccination program for Mwanza Region from the perspective of the Tanzanian government, assuming that HPV vaccines would be delivered through the Expanded Programme on Immunization (EPI).

**Results:**

Total economic project costs for delivering 3 doses of HPV vaccine to 4,211 girls were estimated at about US$349,400 (including a vaccine price of US$5 per dose). Costs per fully-immunized girl were lower for class-based delivery than for age-based delivery. Incremental economic scaled-up costs for class-based vaccination of 50,290 girls in Mwanza Region were estimated at US$1.3 million. Economic scaled-up costs per fully-immunized girl were US$26.41, including HPV vaccine at US$5 per dose. Excluding vaccine costs, vaccine could be delivered at an incremental economic cost of US$3.09 per dose and US$9.76 per fully-immunized girl. Financial scaled-up costs, excluding costs of the vaccine and salaries of existing staff were estimated at US$1.73 per dose.

**Conclusions:**

Project costs of class-based vaccination were found to be below those of age-based vaccination because of more eligible girls being identified and higher vaccine uptake. We estimate that vaccine can be delivered at costs that would make HPV vaccination a very cost-effective intervention. Potentially, integrating HPV vaccine delivery with cost-effective school-based health interventions and a reduction of vaccine price below US$5 per dose would further reduce the costs per fully HPV-immunized girl.

## Background

Cervical cancer is the second most frequent cancer in women worldwide [1]. More than 80% of cervical cancers occur in developing countries [2]. In Tanzania, as in much of sub-Saharan Africa, cervical cancer is the leading cause of female cancer deaths [3,4]. Human papillomaviruses (HPV) are the etiological agents of cervical cancers [5], and vaccines against HPV (types 16 and 18) offer the opportunity to prevent about 70% of cervical cancer cases worldwide [6]. Many high-income countries have national HPV vaccination programs [7], and Tanzania plans to start a national HPV vaccination program in late 2012 [8]. Following the November 2011 decision of the Global Alliance for Vaccines and Immunisations (GAVI) to move towards funding HPV vaccines for eligible countries [9], HPV vaccination could become an option in many countries where HPV vaccines are currently unaffordable.

International studies have estimated that HPV vaccination of young girls would be cost effective in the 72 GAVI-eligible countries [10], and in Eastern Africa in particular [11], if the costs per vaccinated girl were between US$10 and US$25. However, preadolescent and adolescent schoolgirls, the target group for HPV vaccines, are not routinely included in most national immunization programs in sub-Saharan Africa. The costs of delivering HPV vaccines are therefore unknown [11]. To plan for introducing HPV vaccines into national programs and to estimate cost effectiveness, governments and researchers need reliable estimates based on observed local costs.

This costing study was conducted as part of a project in Mwanza Region, Tanzania, which measured the feasibility, uptake, and acceptability of two alternative school-based HPV vaccine delivery strategies using a cluster randomized trial design (NCT ID: NCT01173900) [12]. It aimed to analyze the observed costs of the school-based vaccination project and to explore cost differences between delivering vaccines to rural and urban schools and between the two alternative delivery strategies. It also aimed to estimate incremental costs of a scaled-up vaccination program for Mwanza Region and to calculate the associated costs per fully-immunized girl.

## Methods

First, a top-down cost analysis of the school-based vaccination project was conducted from the project's perspective, using resource use and cost data collected alongside the cluster randomized trial. Second, based on the observed project costs, incremental costs of a scaled-up vaccination program for Mwanza Region were modeled from the perspective of the Tanzanian government, under the assumption that HPV vaccines would be delivered through the Expanded Programme on Immunization (EPI).

## Top-down analysis of project costs

### Study setting and intervention

The HPV vaccination project compared vaccine coverage in 134 primary schools randomly allocated to age-based (girls born in 1998) and class-based (class 6 of primary school in 2010) vaccine delivery in the two districts of Mwanza City and part of neighboring Misungwi district in northwest Tanzania. School-based vaccination was selected for the study because it has the best chance of achieving high coverage in Tanzania [12], where the government has a universal primary education policy and more than 80% of girls attend primary school (87% in urban regions, 80% in rural regions) [13]. In 2010, about 26% of the Tanzanian population were estimated to be living in urban areas and urbanization was rapidly progressing with an annual urban population growth rate of 4.5% [14].

The project was carried out by the Mwanza Intervention Trials Unit (MITU) of the National Institute for Medical Research (NIMR), in collaboration with the London School of Hygiene and Tropical Medicine (LSHTM). (Additional file 1, Table S1 provides more details on the main activities of the intervention.) Ethical approval was obtained from the ethics committees of the Tanzanian Medical Research Coordinating Committee and the London School of Hygiene and Tropical Medicine. More details on consent procedures and refusal rates are presented in Watson-Jones *et al*. [12] and Remes *et al*. [15].

During the preparation phase, 242 schools were mapped and the number of girls in each was documented. Subsequently, 134 schools (60 rural and 74 urban) were randomly selected for vaccination, and then randomly allocated to either age-based or class-based vaccine delivery. Information about HPV vaccination was provided through school, parent and community meetings, leaflet and poster distribution, radio messages and community drama troupes. Two 2-day training sessions were held to train 84 health workers from 42 health facilities in cervical cancer, HPV vaccination, cold-chain logistics and in management and reporting of adverse events.

The quadrivalent (HPV 6, 11, 16 and 18) vaccine (Merck & Co.) was donated by Axios Healthcare Development, Cleveland, Ohio, USA (GARDASIL^® ^Access Programme). The project covered costs of clearance and shipment of vaccines from arrival in Dar es Salaam to Mwanza. Vaccines were stored at MITU in three refrigerators under the supervision of a pharmacist. Between August 2010 and July 2011, eligible girls were offered three doses of vaccine during four vaccination rounds. On vaccination days, project vehicles took MITU supervisory nurses, EPI-trained nurses from health facilities that covered the selected schools, vaccines, and necessary supplies to the schools according to a prearranged timetable. After vaccination days, girls who missed vaccination at schools were given the opportunity to be vaccinated at health facilities during a window period of 2 to 4 weeks. At the end of the window, remaining vaccines were returned to MITU because of cold storage capacity constraints at the health facilities.

Materials used during vaccination were also returned to MITU and incinerated. International and local MITU staff were involved in administration, coordination and supervision of the project. All activities and project inputs related to research, including a proportion of the preparation costs, were excluded from the cost analysis (see Additional file 1, Table S1).

### Data collection and analysis

Full financial and economic costs of running the project in 2010 and the first two quarters in 2011 were estimated using adapted versions of established costing guidelines [16,17]. Financial costs represent actual expenditures on goods and services, while economic costs represent the economic value of all resources used including costs of goods and services that were not paid for by the project (for example, vaccines donated by Axios Healthcare Development).

Cost data were collected from the financial departments of the relevant institutions (MITU, NIMR, and LSHTM) by a researcher (WQ) during two visits to Mwanza in November 2010 and April 2011. Interviews were conducted with all relevant project staff members, as well as with regional and district EPI staff. Logbooks of project cars were evaluated to determine distances travelled. Vaccination of girls was observed by WQ during the second round of vaccination at four schools to record the time that nurses spent on vaccination activities at different types of school. During the third round of vaccination, MITU nurses recorded the time spent at schools and travel times between MITU, health facilities and schools for 40 schools. These data were complemented by time data recorded in logbooks of project cars to estimate the proportion of total time that MITU nurses spent for procurement and vaccination for each type of school (24% of total time for age-based urban, 22% for age-based rural, 28% for class-based urban, and 26% for class-based rural schools). In addition, vehicle logbooks were used to determine the proportion of total km travelled for delivering vaccines to rural and urban schools (75% and 25% of total km, respectively).

All cost data were distributed into five broad categories: (I) personnel, (II) other recurrent (including clinic consumables, communication, and so on), (III) allowances, (IV) transport, and (V) other capital costs for equipment. Annual capital costs were estimated based on replacement costs (for example, of comparable new vehicles or refrigerators) and assuming a life expectancy of 10 years for vehicles and refrigerators, and 5 years for other electronic equipment. Annual financial costs were calculated through straight-line depreciation, while annual economic costs were calculated using a 3% discount rate [18]. An estimate of the average running cost per km of project cars (incl. driver salary, fuel, insurance, repairs) was available from the MITU financial department.

Costs of personnel were assigned to project activities based on staff estimates. Time inputs from government employees contributing to the project (that is, health facility nurses, teachers, other EPI staff) were included under economic costs. Other recurrent costs (for example, vaccination materials and allowances) could be directly attributed to the relevant activities based on information provided in the project management accounts. As vaccines were donated, financial costs exclude costs of the vaccine. Economic costs assume a price per dose of HPV vaccine of US$5 [19]. In some cases, information from the principal investigator, MITU staff, or detailed financial records of the accounting department allowed assignment of costs. Transport costs were allocated to project activities on the basis of the km attributed to each project activity. Other capital costs for equipment (for example, refrigerators, cold boxes) could be directly attributed to relevant activities. Building costs and overheads (for example, housekeeping, water, and electricity except for refrigerators) were not considered.

Costs of training and social mobilization/information, education, communication (IEC) were treated as start-up costs and annuitized over 5 years. All cost data were adjusted to the year 2011 using country-specific GDP deflators [20], and were converted to US$ using average 2011 exchange rates [21].

Average financial and economic costs per fully-immunized girl, that is, per girl who received three doses of vaccine, were calculated by delivery strategy (age-based or class-based) and school location (rural or urban) using a two-step approach. First, total project costs for preparations, social mobilization/IEC, training, administration/supervision, and for most cost elements of procurement and vaccination, were assigned to schools by delivery strategy and school location. Cold storage, recurrent costs of procurement and vaccination, and waste management were allocated to schools on the basis of the average number of doses delivered in each type of school. Second, costs per school were divided by the average number of fully-immunized girls per school. Table 1 presents the number of girls per school by delivery strategy and school location, and compares these numbers to those expected in a regional vaccination program (see below).

**Table 1:** Number of schools and girls (total and per school) by delivery strategy and school location in the Mwanza Vaccination Project and in a scaled-up regional program

	Class-based			Age-based			All schools
			
	Urban	Rural	Total	Urban	Rural	Total	
Vaccination project^a^							
Vaccinated schools	36	30	66	34	30	64	130
Eligible girls (class 6, age 12)^a^	1,924	1,428	3,352	1,186	994	2,180	5,532
Fully-immunized girls							
Total number	1,461	1,178	2,639	795	777	1,572	4,211
Average per school	40.6	39.3	40.0	23.4	25.9	24.6	32.4
Regional program^b^							
Vaccinated schools	190	1,042	1,232	190	1,042	1,232	N/A
Eligible girls (class 4, age 10)	9,855	54,046	63,901	10,244	56,178	66,422	N/A
Fully-immunized girls							
Total number (estimated)^c^	7,756	42,534	50,290	7,386	40,505	47,890	N/A
Average per school	40.8	40.8	40.8	38.9	38.9	38.9	N/A

^a^Based on [12], class-based is for class-year 6, age-based is for girls born in 1998.

^b^Based on [22], class-based is for class-year 4, which is a potential target group for the national human papillomavirus (HPV) vaccination program [8]. Age-based is for 10-year-old girls, which is the age group most closely corresponding to class 4.

The number of fully-immunized girls assumes coverage rates as found in the Mwanza HPV Vaccination project for the class-based and age-based arms, respectively. The two Mwanza City districts are urban, the six other districts of Mwanza Region are rural.

### Incremental costs of a scaled-up regional vaccination program

A model was constructed in order to estimate the incremental financial and economic costs of a scaled-up vaccination program in Mwanza Region using an ingredients approach. Reflecting the plans of the Tanzanian government [8], the model estimates the additional costs of a class-based delivery program targeting girls in class 4 of primary school. Constructing the model involved two steps: first, quantities of necessary resources were estimated on the basis of experiences gathered during the school-based vaccination project, taking into account information from interviews with regional and district EPI staff about service delivery within routine EPI programs, statistics from the Ministry of Education and Vocational Training (MOEVT) [22], and assumptions from a WHO planning and costing tool for estimating costs of introducing HPV vaccines [23]. (Additional file 1, Table S2 provides an overview of the cost categories, inputs included, and sources of information.) Second, unit costs or prices were assigned to the necessary inputs, using data from the analysis of project costs, the EPI comprehensive multi-year plan (CMYP) for Tanzania [8], and the WHO planning and costing tool [23]. (A list of unit costs used in the model can be obtained from the authors upon request.)

The model calculated total costs for Mwanza Region and per fully-immunized girl by multiplying the assumed resources per vaccinated school and girl by the estimated unit costs. First, costs were estimated for a 'base case scenario' using a number of 'base case' assumptions, which are shown in Table 2. Subsequently, the influence of alternative assumptions for critical input parameters was tested through sensitivity analyses. Again, financial costs exclude the costs of HPV vaccine, while economic costs include US$5 per dose.

**Table 2:** Selected input parameters for modeling of a scaled-up vaccination program in Mwanza Region

	Base case	Low cost	High cost
General:			
Number of vaccination rounds	4	3	Base case
Capital discount rate	3%	0%	8%
Life expectancy:			
Vehicles	10 years	15 years	5 years
Refrigerators and cold rooms	10 years	15 years	5 years
Sensitization	5 years	7 years	1 year
Training	5 years	7 years	1 year
Cold storage:			
Cold storage equipment^a^	Incremental volume	Base case	Incremental equipment
Procurement:			
Additional vaccine distribution trips	3	1	4
Km per health facility per vaccination round, urban districts	43	30	50
Km per health facility per vaccination round, rural districts	73	50	90
Wastage rate (vaccines and materials)	5%	1%	10%
Vaccination:			
Number of eligible girls per school^b^	52	60	40
Coverage rates^c ^for dose 1	86%	95%	75%
Coverage rates^c ^for dose 2	84%	90%	65%
Coverage rates^c ^for dose 3	79%	85%	55%
Girls immunized in health facilities (%)	4%		
Unit costs:			
Vaccine price	US$5	US$0	Base case
Vaccine clearance and shipment to regional level (costs/dose)	US$0.39	US$0.20	Base case

^a^Incremental volume refers to estimating the costs of the storage volume required for storing the estimated number of vaccine doses. Incremental equipment refers to the necessary increase in cold storage equipment according to interviews with the district cold chain coordinators in Mwanza and Misungwi districts.

^b^The number of eligible girls per school is varied by increasing/decreasing the total number of eligible girls in Mwanza Region.

^c^As percentage of total cohort of eligible girls.

Base case assumptions usually reflect findings from the project cost analysis, interviews conducted with regional and district EPI staff, a standard discount rate [18], numbers of girls from MOEVT statistics, and, for economic costs, a price per dose of HPV vaccine of US$5 [19]. Low cost assumptions assume only three vaccination rounds, a longer life expectancy of capital equipment as well as of sensitization and training, fewer additional or shorter distribution trips, higher coverage rates (as in existing EPI programs [24]), lower unit costs for the shipment of vaccines to the regional EPI office, and they exclude the costs of HPV vaccine. High cost assumptions use a discount rate as published by the Bank of Tanzania [25], shorter equipment life expectancies, and lower coverage rates.

Univariate and multivariate sensitivity analyses (SA) were carried out using the 'high cost' and 'low cost' assumptions. For the univariate sensitivity analysis, one model parameter was varied at a time with all other parameters kept constant. For the multivariate SA, all variables were simultaneously set to the 'low cost' assumptions or 'high cost' assumptions, respectively, in order to generate the largest possible range of costs in an analysis of extremes [26].

## Results

### Analysis of project costs

#### Total project costs

**Figure 1:**
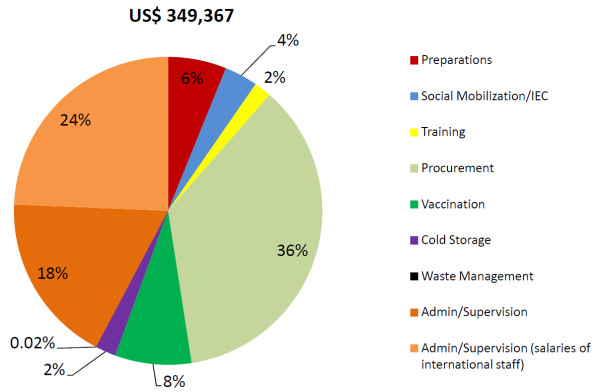
**Contribution of project activities to total economic project costs (year 2011, US$)**.

Total economic project costs for delivering 3 doses of HPV vaccine to 4,211 girls in Mwanza City and Misungwi district (excluding research related activities) were estimated to be US$349,000 (Figure 1). (Data for financial and economic costs and for project costs by cost category are available in Additional file 1, Table S3.) Administration/supervision accounted for 42% of total project costs. Procurement accounted for 36% of total costs or US$126,000, of which 53% were related to the costs of HPV vaccines (assuming a price of US$5 per dose). Costs of social mobilization/IEC and training, which were annuitized over 5 years, accounted for only US$12,200 (3.5%) and US$6,400 (1.8%), respectively.

### Project costs per fully-immunized girl

**Figure 2:**
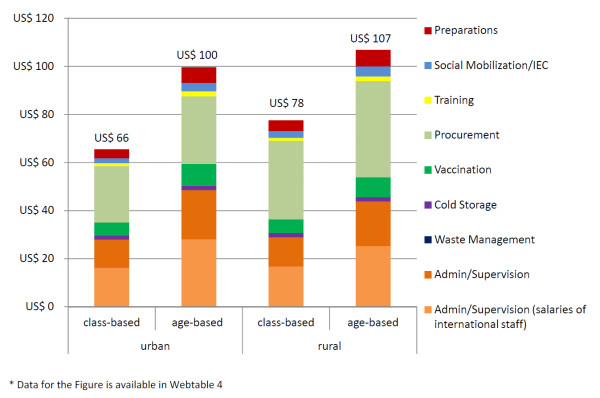
**Project economic costs (year 2011 US$) per fully-immunized girl by school location and vaccination strategy**. Data for the Figure are available in Additional file 1, Table S4.

Figure 2 presents total economic project costs per fully-immunized girl vaccinated with either age-based or class-based delivery by location of school. The number of vaccinated girls was much higher at class-based schools than at age-based schools (Table 1). Total costs per fully-immunized girl at urban schools were US$66 for class-based delivery and US$100 for age-based delivery. Costs at rural schools were US$78 for class-based delivery and US$107 for age-based delivery. Procurement costs, including the costs of transporting vaccines to schools, were considerably higher at rural schools than at urban schools.

### Costs of a scaled-up regional vaccination program

#### Total program costs

Table 3 shows the estimated incremental financial and economic costs for a scaled-up vaccination program in Mwanza Region. Incremental financial costs of delivering 3 doses of HPV vaccine through class-based delivery (class 4 of primary school) to 50,290 girls were estimated to be US$276,000, while economic costs (including vaccine costs at a price of US$5 per dose and the time inputs of existing staff) were estimated to be US$1.3 million. The costs of procurement, including the costs of vaccines and supplies, shipment of vaccines to regional level, transport to districts and distribution to health facilities, account for the largest share of financial and economic costs (46 and 73%, respectively). The costs of vaccination, including the costs of nurses' and teachers' time as well as allowances, account for 17% of economic costs. Administration/supervision accounted for only 4.5% of economic costs.

**Table 3:** Scaled-up class-based total costs of a human papillomavirus (HPV) vaccination program in Mwanza Region (in 2011 US$)*

	Financial costs		Economic costs	
		
Program element	US$	%	US$	%
1. Social mobilization/IEC (annuitized):				
1.1. Meetings (teachers, parents/pupils)	15,656	5.7%	23,504	1.8%
1.2. Campaign (radio, cultural troops)	3,254	1.2%	3,597	0.3%
1.3. Material development	20	0.0%	284	0.0%
Subtotal social mobilization/IEC	18,930	6.9%	27,385	2.1%
2. Training (annuitized):				
2.1. District staff training	1,004	0.4%	2,472	0.2%
2.2. Health facility staff training	8,144	3.0%	21,954	1.7%
2.3. Material development	0	0.0%	524	0.0%
Subtotal training	9,148	3.3%	24,950	1.9%
3. Cold storage:				
3.1. Regional level cold storage	111	0.0%	126	0.0%
3.2. District level cold storage	1,371	0.5%	1,596	0.1%
3.3. Health facility cold storage	13,536	4.9%	14,551	1.1%
Subtotal cold storage	15,018	5.4%	16,273	1.2%
4. Procurement:				
4.1. Vaccines and supplies	19,162	6.9%	856,264	64.5%
4.2. Shipment to regional level	65,116	23.6%	65,116	4.9%
4.3. Transport to districts	2,923	1.1%	4,048	0.3%
4.4. Distribution to health facilities	39,603	14.4%	46,688	3.5%
Subtotal procurement	126,805	46.0%	972,115	73.2%
5. Vaccination:				
5.1. School vaccination	62,992	22.8%	221,044	16.6%
5.2. Health facility vaccination	0	0.0%	5,247	0.4%
Subtotal vaccination	62,992	22.8%	226,291	17.0%
6. Waste management:				
Subtotal waste management	1,020	0.4%	1,020	0.1%
7. Admin/supervision:				
6.1. Regional level supervision	1,026	0.4%	2,047	0.2%
6.2. District level supervision	40,869	14.8%	57,955	4.4%
Subtotal admin/supervision	41,894	15.2%	60,002	4.5%
Overall total	275,807	100%	1,328,037	100%

^a^Costs are for delivering 3 doses of HPV vaccine through class-based delivery (year 4 of primary school) to 50,290 girls in Mwanza Region. Financial costs exclude the costs of the vaccine itself, which will be donated to the Tanzanian government at least during the first years of the national vaccination program [8]. Economic costs include a vaccine price of US$5 per dose.

IEC = information, education, communication.

### Costs per fully-immunized girl and sensitivity analysis

Under base case assumptions for input parameters, the incremental economic scaled-up costs per fully-immunized girl through class-based delivery in Mwanza Region, including the cost of vaccine, were estimated at US$26.41. (Data for urban and rural schools and a comparison with project costs are presented in Additional file 1, Table S5 and Additional file 2, Figure S1.) The distribution of costs across program elements and the relationship between financial and economic costs mirrored the results presented in Table 3. Financial costs per fully-immunized girl, excluding costs of the vaccine and salaries of existing staff, were estimated at US$5.48 or US$1.73 per dose.

**Figure 3:**
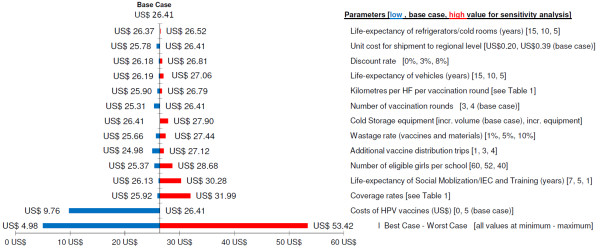
**Scaled-up class-based vaccination program: sensitivity analysis of incremental economic costs per fully-immunized girl (2011 US$)**.

Univariate sensitivity analysis (SA) showed that excluding costs of HPV vaccines would reduce the costs per fully-immunized girl to US$9.76 and a cost per dose given of US$3.09. At 55% coverage for the third dose of HPV vaccine, costs per fully-immunized girl would increase to US$31.99 (Figure 3). Repeating sensitization/IEC and training every year would lead to increased costs of US$30.28, although some of these higher costs might be offset by an increase in coverage. The number of eligible girls per school was also an important determinant of costs. The impact of most other model input parameters on costs was estimated to be small. Alternative assumptions for life expectancy of vehicles and cold-storage equipment did not have a strong influence on estimated costs. Even if life expectancy of cold-storage equipment and vehicles were only 5 years, costs would not considerably increase (Figure 3). Multivariate SA showed that costs can increase more than twice from the base case scenario to about US$53, if all model parameters are set to the alternative high cost assumptions.

## Discussion

The World Health Organization recommends the inclusion of HPV vaccination in national immunization programs for girls 9 to 13 years old if (1) prevention of cervical cancer is a public health priority, (2) the introduction is programmatically feasible, (3) sustained financing can be secured, and (4) the cost effectiveness is considered [27]. However, without detailed analyses of costs based on observed data collected in countries, it is impossible to reliably estimate cost effectiveness or to assess whether sustained financing will be available for HPV vaccination. In sub-Saharan Africa, where more than 1 million cancer cases could be averted through HPV vaccination of 10 consecutive birth cohorts of girls [28], empirical information about the delivery costs of vaccines to young adolescent girls was unavailable until very recently [11,29].

This is one of the first studies to report detailed resource-use-based cost estimates for delivering HPV vaccines to schoolgirls in sub-Saharan Africa. Using data collected alongside a cluster randomized trial, our project cost analysis demonstrated large differences in the costs of delivering HPV vaccines to girls between age-based and class-based delivery. In addition, modeled costs of a scaled-up vaccination program for Mwanza Region in Tanzania showed that three doses of HPV vaccine, when excluding costs of the vaccine itself, could be delivered to girls at an incremental economic cost of US$3.09 per dose and about US$10 per fully-immunized girl. These estimates are similar to the US$3.15 per dose that have been reported by PATH and the national EPI program for a school-based HPV vaccination program in Uganda [29], but significantly higher than estimated for the costs of delivering new childhood vaccines (also excluding vaccine costs), such as pneumococcal or rotavirus vaccines in Kenya (US$1.06) [30], Uganda (US$0.34) [31], or Malawi (US$0.35) [32].

Our study has a number of limitations. First, despite our high reliance on observed data, the analysis of project costs was based on estimates and extrapolations for certain inputs. Personnel time was allocated to different activities of the intervention on the basis of staff interviews, which are a known source of uncertainty [33], although prospective time sheets (from the third round of vaccination), which are a better source of information, were used to allocate costs between procurement and vaccination. Vehicle logbooks were used for the allocation of transport costs but detailed records for the allocation between rural and urban schools were only consistently available for vaccination round three. However, these allocations would not be expected to change over time since the same schools were visited in each round. Furthermore, project costs are not representative of how much it would cost to deliver HPV vaccines within a national immunization program: the project delivered HPV vaccines through a vertical system specifically set up for the intervention. Such systems imply high costs for administration/supervision, transport, and a dedicated cold chain. Procurement costs in the project were particularly high for rural schools as vehicles always had to travel from Mwanza City to the rural district. In the scaled-up program, the difference in procurement costs between urban and rural schools was estimated to be much smaller as vaccines would be delivered from the district EPI office (see Additional file 2, Figure S1). In addition, salary costs of international staff accounted for about 18% of project costs (Figure 1). If they were replaced by their local equivalents, this would result in a reduction in total project costs by almost 20%.

Second, our scaled-up cost estimates, which aimed to overcome the problems of the project cost analysis by adjusting the costs for administration/supervision, transport and cold chain, have other limitations related to the adopted ingredients approach for modeling of costs. National level costs (such as administrative and supervision costs) were excluded, although these cost categories can be important; and the ingredients approach may underestimate inefficiencies. For example, for cold storage costs, the incremental costs of HPV vaccines were calculated as the costs of the additional volume required for storing the vaccines. However, if the introduction of HPV vaccines required an increase in the number of refrigerators or cold rooms, which would not be used during non-HPV-vaccination months, the incremental costs would be much higher. For cold storage, such a scenario was tested in the sensitivity analysis, which revealed that increasing the number of refrigerators in Mwanza Region to what district cold chain coordinators deemed necessary for adding HPV vaccination would increase the costs per fully-immunized girl by about 6%, that is, from US$26.41 to US$27.90 (Figure 3).

Third, our model calculated scaled-up costs based on experiences gathered over the course of the research project and adjusted observed costs based on assumptions derived from interviews with EPI staff in Mwanza City and Misungwi District. The model assumed that similarly high coverage rates would be achieved in a regional program as in the research project; that distances travelled from district stores to health facilities would be similar for all rural districts as those in Misungwi District; and that wastage rates of vaccines and materials would be similar to those in existing EPI programs [24]. However, the influence of alternative assumptions was tested in sensitivity analyses (Figure 3), which showed that except for the unlikely case of the full worst case scenario, estimated costs would be relatively robust.

Despite these caveats, our research has major implications for policymakers and researchers. Firstly, in our project cost analysis, we found that costs per fully-immunized schoolgirl of delivering three doses of HPV vaccines using a class-based delivery strategy were about one-third below those of using age-based delivery, although the costs per school reached were not higher for age-based delivery (see Additional file 3, Figure S2). The reason for the lower costs per fully-immunized schoolgirl of class-based delivery in the project was the substantially higher number of girls vaccinated at class-based schools due to more eligible girls being identified and higher vaccine coverage (Table 1 and Watson-Jones *et al*. [12]). In a scaled-up regional program, which is likely to target a different group of girls (class 4 instead of class 6), the number of eligible girls per school would be similar under age-based and class-based delivery (Table 1). However, targeting a different age group may have further cost and effectiveness implications, which need to be considered. About 99% of girls in class 4 are in the target group for vaccination (between 9 and 13 years of age) [22] and are less likely to ever have had sex before [13,34] than girls in class 6, where almost 20% are 13 years and older. However, as the full duration of protection offered by HPV vaccines still remains uncertain [35] (even if recent evidence has confirmed vaccine efficacy of 8.4 years [36] and modeling results suggest 20 years protection [37]), a trade off might exist between targeting girls early in life (prior to their sexual debut) and ensuring that they are still protected later in life. Furthermore, earlier vaccination implies a requirement for earlier booster doses, which may increase costs.

Secondly, we estimated scaled-up costs of US$26 per girl fully immunized against HPV. Even when excluding vaccine costs (see Figure 3), US$10 is required for delivering vaccines to schoolgirls. It is essential that these costs be adequately budgeted during vaccine introduction, in particular because a previous study found that system costs (social mobilization/IEC, training, cold storage, transport) were not covered by GAVI Alliance introduction grants during the introduction of DTwP-hepatitis B-HiB vaccine in Ethiopia [38]. Furthermore, we found that the costs of a school-based vaccination program, when excluding costs of the vaccine, are mostly driven by the costs of reaching schools, for social mobilization/IEC, procurement and vaccination. Consequently, the marginal costs per fully HPV-immunized girl and social mobilization costs could potentially be reduced if HPV vaccines were delivered together with other school-based health interventions, such as tetanus toxoid (TT) vaccination, vitamin A supplementation or deworming with antihelminthics [29,39] and/or reproductive health interventions. In addition, if recent findings are confirmed that two-dose HPV vaccine regimens may be effective for cervical cancer prevention [40], costs per fully-immunized girl can be expected to drop by about 30%.

Thirdly, our results should be incorporated into future cost-effectiveness analyses of HPV vaccines in Eastern Africa. Our model of a scaled-up regional HPV vaccination program estimated that costs per fully-immunized girl would be about US$26 (see Figure 3), when including a vaccine price of US$5 per dose [19]. Results of existing cost-effectiveness studies [11,41] suggest that HPV vaccination at US$25 per fully-immunized girl would be very cost effective in all countries of Eastern Africa: at US$25 purchasing power parities (PPP) per vaccinated girl, the costs per life-year saved (Campos *et al*. [11]) or per disability adjusted life-year (DALY) averted (Kim *et al*. [41]) would be below the per capita income in these countries. Of course, if HPV vaccine prices drop below US$5 and vaccines are delivered together with other school-based health interventions (see above), cost effectiveness would further increase. Currently, the GAVI decision to support the introduction of HPV vaccines in developing countries [9] and the donation offer to the Tanzanian government of 2 million doses of HPV vaccine [8] present an unprecedented opportunity for the introduction of this life-saving intervention.

## Conclusions

The introduction of HPV vaccination in sub-Saharan Africa offers a new opportunity for cervical cancer control. Our cost estimates suggest that the vaccine can be delivered at costs that would make HPV vaccination a very cost-effective intervention [11]. Potentially, delivering HPV vaccines together with other cost-effective school-based health interventions and a reduction of the vaccine price below US$5 per dose would lead to lower costs and higher cost effectiveness. Furthermore, if two-dose HPV vaccine regimens were effective for cervical cancer prevention [40], costs per fully-immunized girl would drop by about 30%.

## Competing interests

DW-J has received grant support through LSHTM from GlaxoSmithKline Biologicals. WJE's partner works for GlaxoSmithKline. There are no other conflicts of interest.

## Authors' contributions

DW-J conceptualized the study, which was then designed in close collaboration by all authors. WQ, JC, SS, SK, and DW-J assured data collection in Tanzania. WQ, FT-P, WJE, RHu, DAR, RHa, and DW-J analyzed and interpreted the data. WQ drafted the manuscript. All authors critically revised and approved the final manuscript.

## Pre-publication history

The pre-publication history for this paper can be accessed here:

http://www.biomedcentral.com/1741-7015/10/137/prepub

## Supplementary Material

Additional file 1 **Additional tables**. Table S1: activities performed during the Mwanza human papillomavirus (HPV) vaccination project. Table S2: program elements and included inputs for modeling of scaled-up regional vaccination program. Table S3a: project costs by activity: total financial and economic costs (year 2011 US$) for vaccination of 4,211 girls. Table S3b: project costs by cost category: total financial and economic costs (year 2011 US$) for vaccination of 4,211 girls. Table S4: data for Figure 2: project economic costs (year 2011 US$) per fully-immunized girl by school location and vaccination strategy. Table S5: data for Additional file 2, Figure S1: economic costs (year 2011 US$) per fully-immunized girl in a scaled-up regional vaccination program and in the Mwanza Vaccine Project by school location. Click here for file

Additional file 2 **Figure S1**. Economic costs (year 2011 US$) per fully-immunized girl in a scaled-up regional vaccination program and in the Mwanza Vaccine Project (class-based delivery strategy) by school location. Click here for file

Additional file 3 **Figure S2**. Economic costs (year 2011 US$) per vaccinated school in the Mwanza Vaccine Project by school location and vaccination strategy. Click here for file
