# Supplementary material for: Costs of delivering human papillomavirus vaccination to schoolgirls in Mwanza Region, Tanzania
Source: BMC Med. 2012 Nov 13;10:137. doi: 10.1186/1741-7015-10-137 (PMC3520755; doi:10.1186/1741-7015-10-137)
Supplement: Additional file 1 — Additional tables. Table S1: activities performed during the Mwanza human papillomavirus (HPV) vaccination project. Table S2: program elements and included inputs for modeling of scaled-up regional vaccination program. Table S3a: project costs by activity: total financial and economic costs (year 2011 US$) for vaccination of 4,211 girls. Table S3b: project costs by cost category: total financial and economic costs (year 2011 US$) for vaccination of 4,211 girls. Table S4: data for Figure 2: project economic costs (year 2011 US$) per fully-immunized girl by school location and vaccination strategy. Table S5: data for Additional file 2, Figure S1: economic costs (year 2011 US$) per fully-immunized girl in a scaled-up regional vaccination program and in the Mwanza Vaccine Project by school location. [file 1741-7015-10-137-S1.DOC]

## Additional file 1

Table S1: Activities performed during the Mwanza HPV vaccination project

| Intervention category | Included activities and inputs |
| --- | --- |
| 1) Preparation | - Mapping of 242 schools to document numbers of girls eligible for vaccination - Selection of 134 schools for vaccination (60 rural, 74 urban) - Visits to health facilities, assessing capacity to assist with vaccination at schools |
| 2) Social Mobilization/IEC  (start-up, life: 5 years) | - Designing of posters and leaflets - 134 meetings with teachers - 134 parent meetings - 13 meetings with religious leaders - 5 meetings with village councils - 4 days mobilisation with community drama troupes - Broadcasting of radio messages (twice for 30 min) |
| 3) Training  (start-up, life: 5 years) | - Preparation of training materials - Two two-day training sessions (one in Mwanza, one in Misungwi): 84 health workers from 42 health facilities were trained |
| 4) Cold Chain Storage | - Storage of vaccines at MITU in three refrigerators (total capacity of 400 l) - One pharmacist who supervised and monitored refrigerators - One freezer (140 l capacity) used to produce ice packs - Four cold boxes and six vaccine carriers used for school visits - Storage of vaccine at health facilities during 4 weeks after vaccination at schools |
| 5) Procurement | - Clearance and shipment of vaccines from arrival in country to Mwanza - US$5 per dose of HPV vaccine (for economic costs) - Necessary supplies (autodisposable syringes, safety boxes, other medical supplies, vaccination cards and records) - Transport of vaccines, supplies, MITU nurses, and EPI nurses to schools by project cars. - Pick-up of vaccines from health facilities after end of vaccination window |
| 6) Vaccination | - Four vaccination rounds between August 2010 and July 2011, 130 schools vaccinated (4 refused, 64 age-based, 66 class-based) - Vaccination at schools by one EPI nurse together with one or two MITU nurses and one or two teachers. - Vaccination at health facilities during two to four weeks following vaccination at schools. |
| 7) Waste Management | - Incineration of used syringes at MITU |
| 8) Administration/ supervision | - MITU project coordinator - Supervision by principal investigator (LSHTM staff) - Administrative support from supplies officer, accountant, administrator, data manager, and IT manager - Supervision by one senior administrator (LSHTM staff) - Supervision by regional EPI staff. |
| 9) Research | - Dedicated research staff (interviewees, researchers) - Time of principal investigator, MITU project coordinator, and MITU nurses spent on research - Preparations costs related to mapping of schools that were not selected for vaccination - 50% of MITU nurses’ time during the third and fourth round of vaccination - A proportion of administration staff was allocated to research based on the percentage of research costs out of total costs or research staff (full time equivalents) out of total staff - Research related equipment, transport, other recurrent |

**Table S2:** Programme Elements and Included Inputs for Modelling of Scaled-up Regional Vaccination Programme

| **Category** | **Program inputs** | **Source/adjustments** |
| --- | --- | --- |
| **1. Mobilisation/IEC** |  |  |
| *1.1. Meetings* |  |  |
| 1.1.1 teachers meetings (one per school) |  | *Project experience* |
| -- personnel | 1 nurse, 11 teachers | 1 EPI nurse instead of 3 MITU staff, teachers as in project |
| -- other recurrent | allowances for nurse and teachers, leaflets, posters, communication | as in project except no transport from project office to school |
| 1.1.2 parents/pupils meetings (one per school) |  | *Project experience* |
| -- personnel | 1 nurse | 1 EPI nurse instead of 3 MITU staff |
| -- other recurrent | allowance for nurse, leaflets, tally sheet, communication | as in project except no transport from project office to school |
| 1.1.3 Materials |  | *Project experience* |
| -- other recurrent | posters, leaflets |  |
| *1.2. Campaign (radio, cultural troops)* |  | *Project experience* |
| 1.2.1 cultural troops | *40 days campaign with cultural troops* | *10 times more days than in project* |
| -- personnel | 1 nurse | 1 EPI nurse instead of MITU nurse |
| -- other recurrent | cultural troop fee, transport, allowance, communication | 10 times more kilometres than in project |
| 1.2.2 Radio messages(45 radio spots) |  | *RCCO interview* |
| -- personnel | preparation time (4 hours of RCCO time) |  |
| -- other recurrent | airtime |  |
| *1.3. Material development* |  | Project experience |
| -- personnel | development time (20 days of RCCO time) | RCCO time instead of project coordinator |
| -- other recurrent | artist fee | Project experience |
|  |  |  |
| **2. Training** |  |  |
| *2.1. District staff training (4 days, one session)* |  | RCCO interview |
| -- personnel | 3 Trainers, 24 Trainees + preparation time | RCCO interview |
| -- other recurrent | allowances, venue hire, catering, invitation letters, training materials | project experience |
| *2.2. Health facility staff training (3 days, 16 sessions)* |  | RCCO/DCCO interviews |
| -- personnel | 3 Trainers, 2 Trainees per health facility + preparation time | DCCO interviews/WHO C4P tool |
| -- other recurrent | allowances, venue hire, catering, invitation letters, training materials | project experience |
| *2.3. Material development* |  |  |
| -- personnel | development time | project experience |
|  | | |
| **3. Cold Storage** |  | |
| *3.1. Regional level* |  |  |
| -- capital costs: equipment | litres of refrigerator capacity required (cold room, generator) | estimated based on vaccine volume |
| -- recurrent costs | electricity | estimated |
| -- capital costs: equipment | litres of refrigerator capacity required, cold boxes | estimated based on vaccine volume |
| -- recurrent costs | electricity | estimated based on WHO product information sheets |
| *3.3 Health facility level* |  |  |
| -- capital costs: equipment | litres of refrigerator capacity, vaccine carriers | estimated based on vaccine volume |
| -- recurrent costs | gas for cooling | estimated based on WHO product information sheets |
|  |  |  |
| **4. Procurement** |  |  |
| *4.1. Supplies* | Syringes, safety boxes, vaccination cards, tally sheets, waste bags | project experience |
| *4.2. Shipment of vaccines to regional level* | Clearance and transport of vaccines from arrival in country to regional level | project experience |
| *4.2 Transport of vaccines and supplies to district* |  |  |
| -- personnel | DCCO pick-up time from regional level | RCCO interview |
| -- other recurrent | Allowances (DCCO and driver), vehicle and driver costs per km | RCCO interview |
| *4.2 Distribution of vaccines and supplies to health facilities* |  |  |
| -- personnel | DCCO time for delivery to health facilities | DCCO interviews |
| -- other recurrent | Allowances (DCCO and driver), vehicle and driver costs per km | DCCO interviews |
|  |  |  |
| **5. Vaccination** |  |  |
| *5.1. School Vaccination* |  | project experience |
| -- personnel (for economic costs) | 1 nurse, 1 teacher (one day per school and round) | 1 EPI nurse (without assistance by 1 or 2 MITU nurses) |
| -- other recurrent | allowances (nurse and teacher) | EPI nurse interviews |
| *5.2. Health Facility Vaccination* |  |  |
| -- personnel | nurse time per girl | EPI nurse observations |
|  |  |  |
| **6. Waste management** |  |  |
| 6.1. other recurrent | needle destruction costs per dose | project experience |
|  |  | |
| **7. Admin/Supervision** |  | |
| *7.1 Regional level Admin/Supervision* |  |  |
| -- personnel (Economic costs) | RCCO time (monthly and supervision visits) | RCCO interview |
| -- other recurrent | allowance for supervision trips (RCCO and driver), costs per km | WHO C4P tool |
| *7.2. District level Admin/Supervision* |  |  |
| -- personnel | DCCO (monitoring vaccine consumption), health facility supervision trips (costs shared with procurement), | DCCO interviews |
| -- other recurrent | Allowances for supervision trips (DCCO, School Health coordinator, driver), costs per km (shared with procurement) | DCCO interviews |

| **Table S3a:** Project Costs by activity: Total financial and economic costs (year 2011 US$) for vaccination of 4,211 Girls | | | | | |
| --- | --- | --- | --- | --- | --- |
|  | Financial Costs | |  | Economic Costs | |
|  | (US$) | (%) |  | (US$) | (%) |
| Preparations | US$ 20,414 | 7.9% |  | US$ 21,412 | 6.1% |
| Social Mobilization/IEC | US$ 10,009 | 3.9% |  | US$ 12,229 | 3.5% |
| Training | US$ 5,219 | 2.0% |  | US$ 6,368 | 1.8% |
| Procurement | US$ 56,840 | 22.0% |  | US$ 126,138 | 36.1% |
| - HPV vaccine (at US$5 per dose) | US$ 0 | 0.0% |  | US$ 67,473 | 19.3% |
| Vaccination | US$ 12,678 | 4.9% |  | US$ 28,010 | 8.0% |
| Cold Storage | US$ 6,646 | 2.6% |  | US$ 7,521 | 2.2% |
| Waste Management | US$ 82 | 0.0% |  | US$ 82 | 0.0% |
| Admin/Supervision | US$ 61,347 | 23.8% |  | US$ 62,555 | 17.9% |
| Admin/Supervision (salaries of international staff) | US$ 85,052 | 32.9% |  | US$ 85,052 | 24.3% |
| Total | US$ 258,286 | 100.0% |  | US$ 349,367 | 100.0% |
|  |  |  |  |  |  |
| **Table S3b:** Project Costs by Cost Category: Total Financial and Economic Costs (year 2011 US$) for Vaccination of 4,211 Girls | | | | | |
|  | Financial Costs | |  | Economic Costs | |
|  | (US$) | (%) |  | (US$) | (%) |
| Personnel | US$ 188,660 | 73.0% |  | US$ 208,192 | 59.6% |
| Allowances | US$ 10,051 | 3.9% |  | US$ 10,274 | 2.9% |
| Other recurrent | US$ 12,563 | 4.9% |  | US$ 80,253 | 23.0% |
| - HPV vaccine (at US$5 per dose) | US$ 0 | 0.0% |  | US$ 67,473 | 19.3% |
| Transport | US$ 44,666 | 17.3% |  | US$ 47,361 | 13.6% |
| Other capital | US$ 2,347 | 0.9% |  | US$ 3,287 | 0.9% |
| Total | US$ 258,286 | 100.0% |  | US$ 349,367 | 100.0% |

| **Table S4:** Data for Figure 2: Project Economic Costs (year 2011 US$) per Fully-immunized Girl by School Location and Vaccination Strategy. | | | | | | | | | |
| --- | --- | --- | --- | --- | --- | --- | --- | --- | --- |
|  |  |  |  |  |  |  |  |  |  |
|  | urban | | | |  | rural | | | |
|  | class-based | % | age-based | % |  | class-based | % | age-based | % |
| Preparations | US$ 3.77 | 6% | US$ 6.54 | 7% |  | US$ 4.55 | 6% | US$ 6.89 | 6% |
| Social Mobilization/IEC | US$ 2.02 | 3% | US$ 3.50 | 4% |  | US$ 2.76 | 4% | US$ 4.18 | 4% |
| Training | US$ 1.21 | 2% | US$ 2.10 | 2% |  | US$ 1.25 | 2% | US$ 1.89 | 2% |
| Cold Storage | US$ 23.45 | 36% | US$ 28.08 | 28% |  | US$ 32.65 | 42% | US$ 40.01 | 37% |
| Procurement | US$ 5.30 | 8% | US$ 9.04 | 9% |  | US$ 5.67 | 7% | US$ 8.23 | 8% |
| Vaccination | US$ 1.78 |  | US$ 1.83 |  |  | US$ 1.76 |  | US$ 1.78 |  |
| Waste Management | US$ 0.02 | 0% | US$ 0.02 | 0% |  | US$ 0.02 | 0% | US$ 0.02 | 0% |
| Admin/Supervision | US$ 11.86 | 18% | US$ 20.58 | 21% |  | US$ 12.25 | 16% | US$ 18.58 | 17% |
| Admin/Supervision (salaries of international staff) | US$ 16.12 | 25% | US$ 27.98 | 28% |  | US$ 16.66 | 21% | US$ 25.26 | 24% |
| Total | US$ 65.53 | 100% | US$ 99.66 | 100% |  | US$ 77.57 | 100% | US$ 106.85 | 100% |

| **Table S5:** Data for Additional file 2, Figure S1: Economic Costs (year 2011 US$) per Fully-immunized Girl in a Scaled-up Regional Vaccination Programme and in the Mwanza Vaccine Project by School Location. | | | | | |
| --- | --- | --- | --- | --- | --- |
|  |  |  |  |  |  |
|  | scaled-up costs | |  | Project costs | |
|  | urban | rural |  | urban | rural |
| Preparations | n/a | n/a |  | US$ 3.8 | US$ 4.5 |
| Social Mobilization/IEC | US$ 0.5 | US$ 0.5 |  | US$ 2.0 | US$ 2.8 |
| Training | US$ 0.3 | US$ 0.5 |  | US$ 1.2 | US$ 1.2 |
| Procurement | US$ 18.7 | US$ 19.4 |  | US$ 23.5 | US$ 32.6 |
| Vaccination | US$ 5.0 | US$ 4.4 |  | US$ 5.3 | US$ 5.7 |
| Cold Storage | US$ 0.2 | US$ 0.3 |  | US$ 1.8 | US$ 1.8 |
| Waste Management | US$ 0.0 | US$ 0.0 |  | US$ 0.0 | US$ 0.0 |
| Admin/Supervision | US$ 0.5 | US$ 1.3 |  | US$ 28.0 | US$ 28.9 |
| Total | US$ 25.3 | US$ 26.6 |  | US$ 65.5 | US$ 77.6 |
