# Supplementary material for: Costs of delivering human papillomavirus vaccination to schoolgirls in Mwanza Region, Tanzania
Source: BMC Med. 2012 Nov 13;10:137. doi: 10.1186/1741-7015-10-137 (PMC3520755; doi:10.1186/1741-7015-10-137)
Supplement: Additional file 2 — Figure S1. Economic costs (year 2011 US$) per fully-immunized girl in a scaled-up regional vaccination program and in the Mwanza Vaccine Project (class-based delivery strategy) by school location. [file 1741-7015-10-137-S2.PDF]

Figure S1: Economic Costs (year 2011 US$) per Fully-immunized Girl in a Scaled-up Regional Vaccination Programme and in the Mwanza Vaccine Project (class-based delivery strategy) by School Location.*

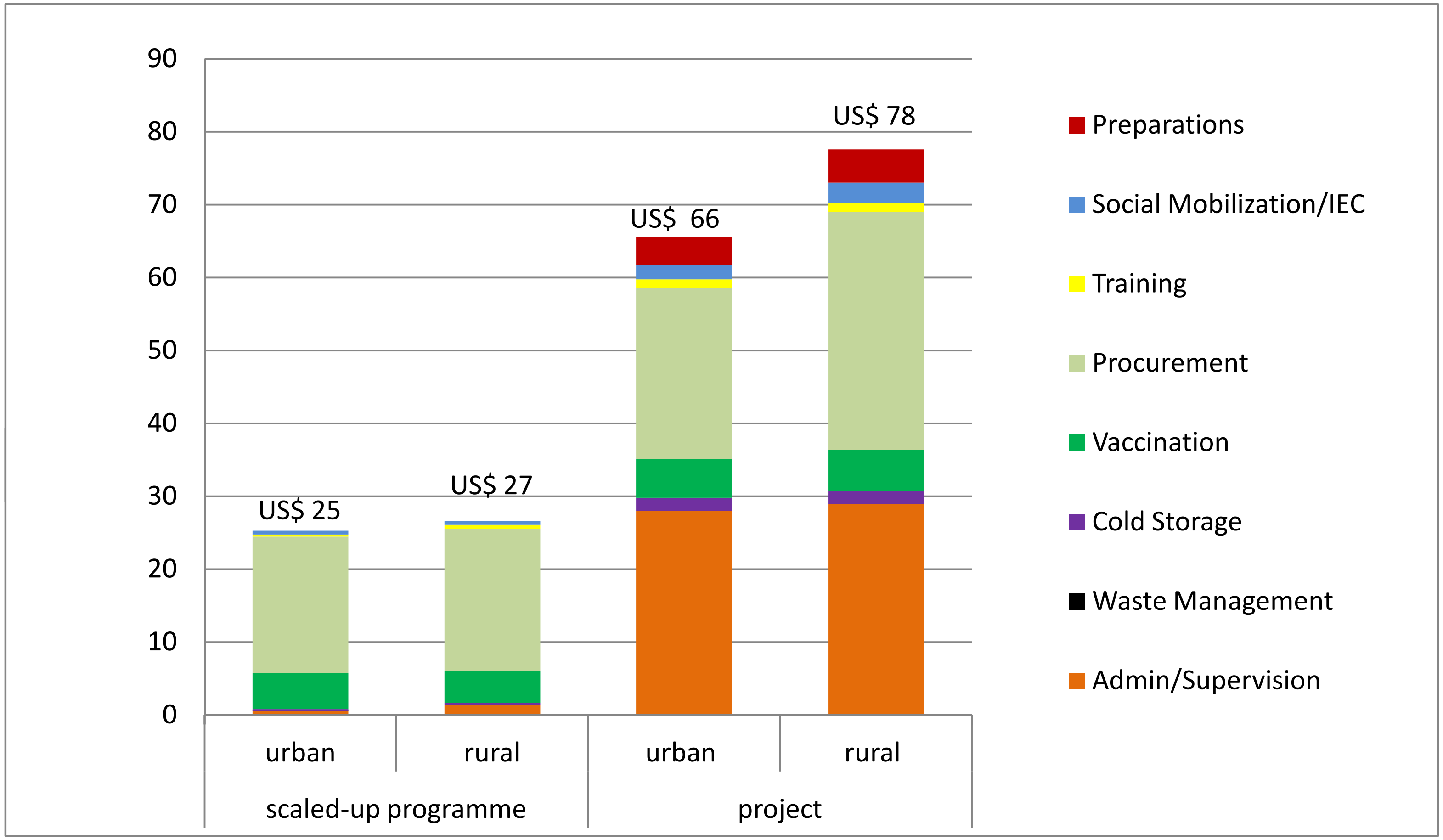

* Data for the figure is provided in Additional file 1, Table S5
