# Supplementary material for: Costs of delivering human papillomavirus vaccination to schoolgirls in Mwanza Region, Tanzania
Source: BMC Med. 2012 Nov 13;10:137. doi: 10.1186/1741-7015-10-137 (PMC3520755; doi:10.1186/1741-7015-10-137)

Figure S2: Economic Costs (year 2011 US$) per Vaccinated School in the Mwanza Vaccine Project by School Location and Vaccination Strategy.

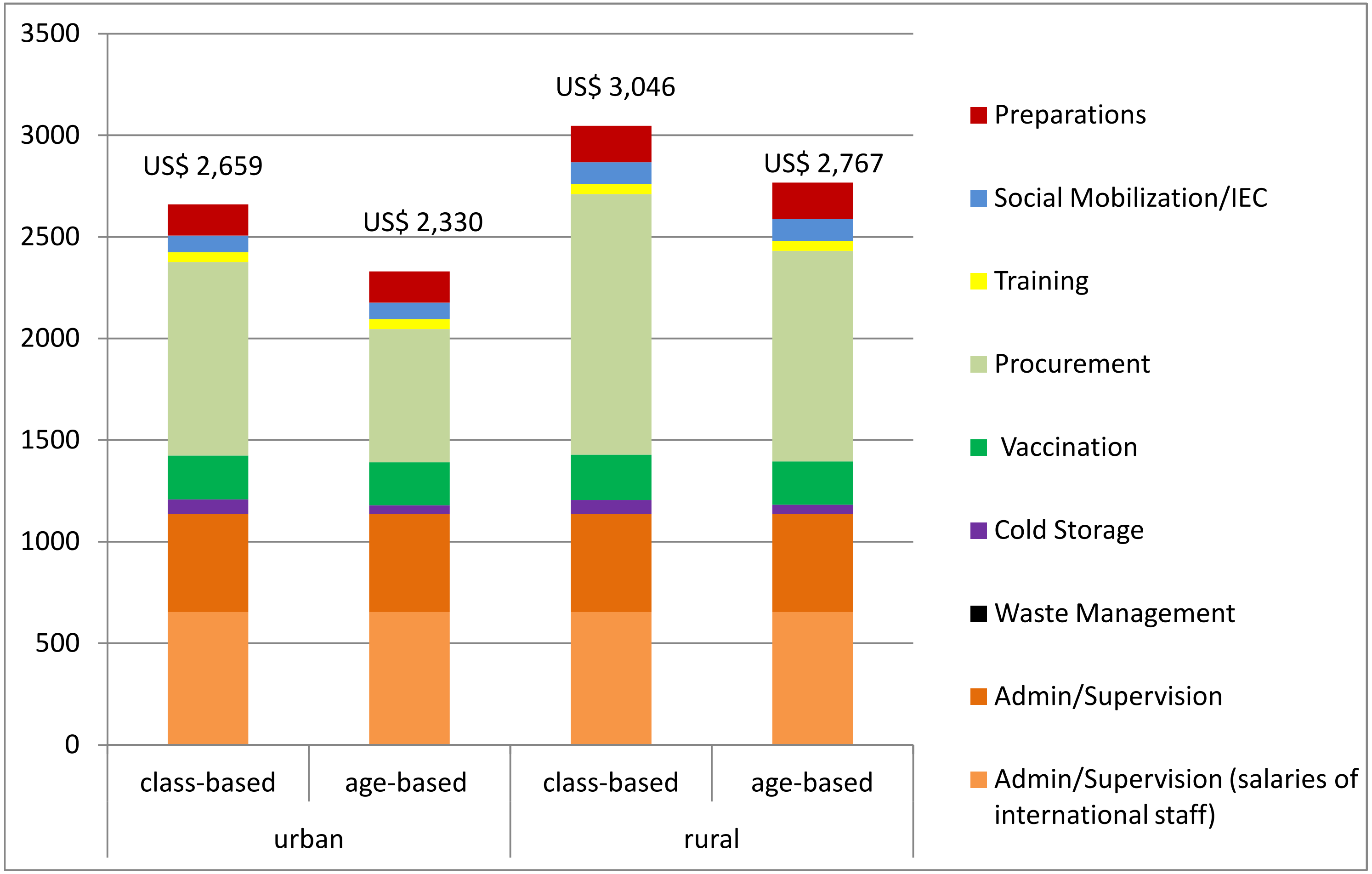

Supplement: Additional file 3 — Figure S2. Economic costs (year 2011 US$) per vaccinated school in the Mwanza Vaccine Project by school location and vaccination strategy. [file 1741-7015-10-137-S3.PDF]
